# Differential patterns of reactive oxygen species and antioxidative mechanisms during atrazine injury and sucrose-induced tolerance in *Arabidopsis thaliana *plantlets

**DOI:** 10.1186/1471-2229-9-28

**Published:** 2009-03-13

**Authors:** Fanny Ramel, Cécile Sulmon, Matthieu Bogard, Ivan Couée, Gwenola Gouesbet

**Affiliations:** 1 Centre National de la Recherche Scientifique, Université de Rennes I, UMR 6553 ECOBIO, Campus de Beaulieu, bâtiment 14A, F-35042 Rennes Cedex, France; 2 INRA, UMR 1095 Génétique, Diversité et Ecophysiologie des Céréales, 234-avenue du Brezet, F-63100 Clermont-Ferrand, France

## Abstract

**Background:**

Besides being essential for plant structure and metabolism, soluble carbohydrates play important roles in stress responses. Sucrose has been shown to confer to Arabidopsis seedlings a high level of tolerance to the herbicide atrazine, which causes reactive oxygen species (ROS) production and oxidative stress. The effects of atrazine and of exogenous sucrose on ROS patterns and ROS-scavenging systems were studied. Simultaneous analysis of ROS contents, expression of ROS-related genes and activities of ROS-scavenging enzymes gave an integrative view of physiological state and detoxifying potential under conditions of sensitivity or tolerance.

**Results:**

Toxicity of atrazine could be related to inefficient activation of singlet oxygen (^1^O_2_) quenching pathways leading to ^1^O_2 _accumulation. Atrazine treatment also increased hydrogen peroxide (H_2_O_2_) content, while reducing gene expressions and enzymatic activities related to two major H_2_O_2_-detoxification pathways. Conversely, sucrose-protected plantlets in the presence of atrazine exhibited efficient ^1^O_2 _quenching, low ^1^O_2 _accumulation and active H_2_O_2_-detoxifying systems.

**Conclusion:**

In conclusion, sucrose protection was in part due to activation of specific ROS scavenging systems with consequent reduction of oxidative damages. Importance of ROS combination and potential interferences of sucrose, xenobiotic and ROS signalling pathways are discussed.

## Background

Although molecular oxygen (O_2_) is used as stable terminal electron acceptor in many essential metabolic processes, its partially reduced or activated forms, singlet oxygen (^1^O_2_), superoxide radical (O_2_^.-^), hydrogen peroxide (H_2_O_2_) and hydroxyl radical (HO^.^), are highly reactive [1]. Overproduction of these reactive oxygen species (ROS) can initiate a variety of autooxidative chain reactions on membrane unsaturated fatty acids, thus yielding lipid hydroperoxides and cascades of events ultimately leading to destruction of organelles and macromolecules [2].

In plants, ROS are continuously produced as byproducts of various metabolic pathways, principally through electron transport chains in chloroplasts and mitochondria, photorespiration in peroxisomes, oxidases and peroxidases [3]. ROS, which also act as signalling molecules, have been shown to affect the expression of multiple genes [2,4], and to be involved in activation and control of various genetic stress-response programs [5].

However, numerous environmental factors such as UV-radiation, high light, drought, low or high temperature, mechanical stress and some xenobiotics disturb the prooxidant-antioxidant balance and lead to irreparable metabolic dysfunctions and cell death [6]. Different classes of herbicides are direct or indirect sources of oxidative damages in plants. The herbicide atrazine, of the triazine family, binds to the D1 protein, which results in inhibition of photosystem II (PSII) by blocking electron transfer to the plastoquinone pool [7], thus leading to production of triplet chlorophyll and ^1^O_2 _[8,9].

Because of widespread use, atrazine is a common contaminant in soils, streams, rivers and lakes [10,11]. The length of water residence time associated with high loading rates results in prolonged exposure of phytoplankton communities to atrazine. Numerous studies have been carried out on the sensitivity of aquatic photosynthetic communities towards atrazine and on effects of this herbicide on reduction of photosynthesis, chlorophyll synthesis, cell growth and nitrogen fixation [12,13]. In the case of wild terrestrial plants, most studies deal with mutations of D1 protein in atrazine-resistant weeds [14], rather than with atrazine-related toxic effects.

Exogenous supply of soluble sugars, particularly sucrose, has been shown to confer to Arabidopsis plantlets a high level of atrazine tolerance [15-17]. Transcriptome profiling revealed that atrazine sensitivity and sucrose-induced atrazine tolerance were associated with important modifications of gene expression related to ROS defence mechanisms, repair mechanisms, signal transduction and cellular communication [18]. Thus, sucrose-induced atrazine tolerance was shown to depend on modifications of gene expression, which to a large extent resulted from combined effects of sucrose and atrazine. This strongly suggested important interactions of sucrose and xenobiotic signalling or of sucrose and ROS signalling, thus resulting in induction of specific transcription factors and in an integrated response to changing environmental conditions [18].

Complex arrays of detoxification mechanisms have been selected in plants against ROS accumulation and toxicity. Biochemical antioxidants, such as ascorbate, glutathione, tocopherol, flavonoids, anthocyanins and carotenoids [19,20], and ROS-scavenging enzymes, such as superoxide dismutase (SOD), ascorbate peroxidase (APX), glutathione peroxidase (GPX) and catalase (CAT) [21-23], are involved in maintaining the redox balance of cells. For example, transgenic plants with enhanced SOD activity exhibit increased tolerance to oxidative stress [22,24,25]. Moreover, Ramel et al. [18] have shown that, during sugar-induced protection against atrazine, expression of several ROS defence-related genes was enhanced.

The present work analyses the relationships between ROS patterns, expression of genes involved in synthesis of antioxidant molecules or antioxidative processes and respective enzyme activities in order to characterize atrazine sensitivity and sucrose-induced tolerance against atrazine-dependent oxidative stress. Atrazine-treated plantlets were found to exhibit an original pattern of ROS with increased levels of ^1^O_2 _and H_2_O_2 _associated with a decrease of O_2_^.- ^content, whereas the protective sucrose-atrazine combination favored accumulation of O_2_^.- ^and H_2_O_2_. These ROS patterns were associated with differences of antioxidant gene expression and enzyme activities, thus suggesting that atrazine injuries might be due to deficient ROS-detoxification mechanisms. The possible interferences of sucrose, xenobiotic and ROS signalling are discussed.

## Results

### Patterns of accumulation of singlet oxygen, superoxide radical and hydrogen peroxide

The transfer of plantlets after 3 weeks of growth to control and treatment media, as described in Methods, was designed to compare plantlets at the same developmental and physiological stages. As previously described in numerous studies of sugar effects in plants, mannitol treatment was used as osmotic control. Moreover, we previously showed that the deleterious effects of atrazine on Arabidopsis plantlets followed the same dose-response curve and the same time dependence in the absence or presence of 80 mM mannitol [16,17]. It was also verified that, in accordance with previous studies [26], exogenous sugar treatment resulted in increased levels of endogenous soluble sugars in Arabidopsis plantlets (data not shown).

**Figure 1:**
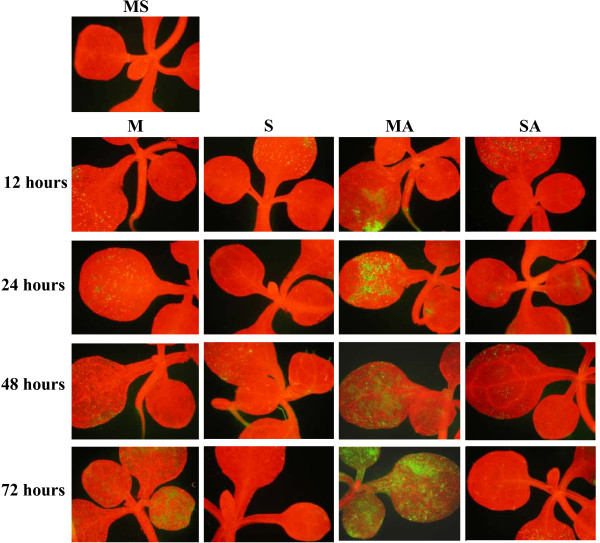
**Visualization of singlet oxygen detected with the SOSG fluorescent probe**. Detections have been done on 3-week-old MS-grown *Arabidopsis thaliana *plantlets subjected to subsequent treatment (12, 24, 48 or 72 hours) with 80 mM mannitol (M), 80 mM sucrose (S), 80 mM mannitol plus 10 μM atrazine (MA) or 80 mM sucrose plus 10 μM atrazine (SA). The fluorescence of SOSG corresponds to the green coloration, while the red color corresponds to chlorophyll autofluorescence. Green fluorescence of roots corresponds to flavonoid and porphyrin autofluorescence. Individual plantlets under the microscope are shown. Quantification of singlet oxygen is presented in Additional file 1.

**Figure 2:**
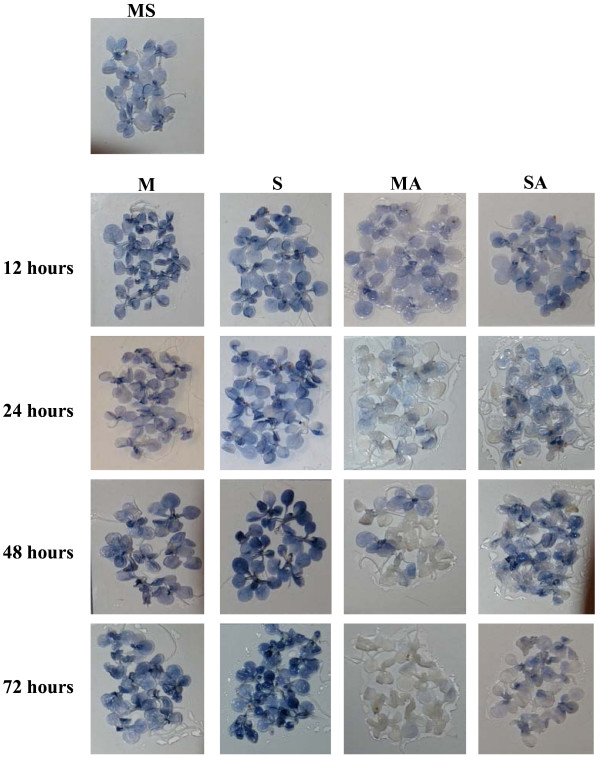
**Visualization of superoxide radical detected by NBT staining**. Detections have been done on 3-week-old MS-grown *Arabidopsis thaliana *plantlets subjected to subsequent treatment (12, 24, 48 or 72 hours) with 80 mM mannitol (M), 80 mM sucrose (S), 80 mM mannitol plus 10 μM atrazine (MA) or 80 mM sucrose plus 10 μM atrazine (SA). Groups of 15 plantlets are shown. Quantification of superoxide radical is presented in Additional file 2.

**Figure 3:**
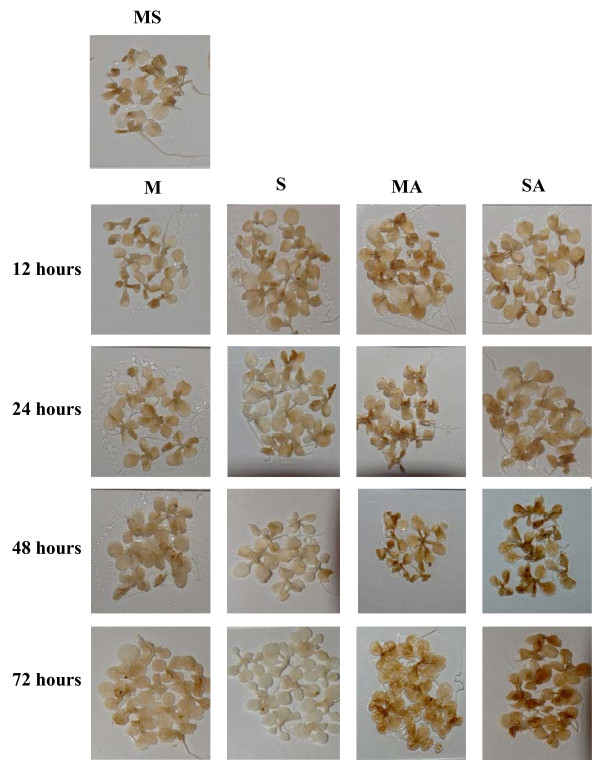
**Visualization of hydrogen peroxide detected by DAB staining**. Detections have been done on 3-week-old MS-grown *Arabidopsis thaliana *plantlets subjected to subsequent treatment (12, 24, 48 or 72 hours) with 80 mM mannitol (M), 80 mM sucrose (S), 80 mM mannitol plus 10 μM atrazine (MA) or 80 mM sucrose plus 10 μM atrazine (SA). Groups of 15 plantlets are shown. Quantification of hydrogen peroxide is presented in Additional file 3.

At the end of treatments, plantlets were specifically stained for singlet oxygen, superoxide radical, and hydrogen peroxide. Hideg et al. [27] described some limitations in the use of vacuum infiltration of ROS probes and reagents with excised leaves or leaf segments from pea, spinach or tobacco. However, vacuum infiltration has been successfully used on whole *Arabidopsis thaliana *plantlets under various experimental conditions [28-30]. Moreover, under the conditions of the present work, whatever the dye used and therefore the ROS detected, the non-stressed plantlets, transferred to 80 mM mannitol or 80 mM sucrose media, presented expected responses related to ROS production (Fig. 1, 2 and 3; Additional files 1, 2 and 3). Plantlets that were transferred for 12 h on mannitol medium presented the same ROS levels as three-week-old plantlets prior to transfer (Fig. 1, 2 and 3; Additional files 1, 2 and 3).

Detection and quantification of singlet oxygen (^1^O_2_) were performed with the specific Singlet Oxygen Sensor Green^® ^reagent [31]. For atrazine-containing treatments (MA and SA), green fluorescence indicating primary events of ^1^O_2 _accumulation was detected in cotyledons as soon as after 12 hours of treatment (Fig. 1 and Additional file 1). Tolerance treatment (SA) maintained a low level of ^1^O_2 _in cotyledons throughout the treatment, while atrazine treatment (MA) strongly increased ^1^O_2 _production in cotyledons and leaves from 12 to 72 hours of treatment. The presence of sucrose in herbicide-containing medium thus appeared to prevent accumulation of ^1^O_2 _generated by atrazine.

Superoxide radical (O_2_^.-^) detection and quantification were performed using the nitroblue tetrazolium (NBT) staining method. The levels of superoxide radical staining after 12 hours of transfer (Fig. 2 and Additional file 2) were quite similar in the absence (M or S) or presence (MA or SA) of 10 μM atrazine. However, the time-course revealed constant levels of O_2_^.- ^in control plantlets (M), while a strong blue coloration appeared in plantlets treated with sucrose (S). This increase was more visible in young leaves. Superoxide radical levels in atrazine-treated plantlets (MA) decreased from 24 hours of treatment. The combination of sucrose plus atrazine (SA) led to an intermediate state with slight coloration maintained in young leaves throughout the treatment. Low levels of O_2_^.-^, relatively to the mannitol control, were also observed when a drop of 10 μM atrazine solution was directly applied to leaf tissue (data not shown).

H_2_O_2 _detection and quantification were performed using the 3,3'-diaminobenzidine (DAB) staining method [32]. Polymerization of DAB, visible as a brown precipitate in the presence of H_2_O_2_, was detected under all conditions. No coloration was observed when infiltration was carried out in the presence of ascorbic acid, thus confirming the H_2_O_2 _specificity of DAB staining, in accordance with previous reports [33-36]. Figure 3 and Additional file 3 summarize the time-course of H_2_O_2 _accumulation. From 24 hours of transfer, control (M) and sucrose-treated (S) plantlets exhibited a much weaker level of H_2_O_2 _than plantlets of atrazine-containing treatments (MA and SA). No variation of H_2_O_2 _accumulation was detected in the presence of mannitol, whereas H_2_O_2 _content decreased in sucrose-treated plantlets. In contrast, atrazine in the absence or presence of sucrose tended to increase progressively H_2_O_2 _levels until 72 hours of treatment. This increase could be detected as early as the fourth hour of atrazine treatment (data not shown). Likewise, an immediate increase of H_2_O_2 _levels was also observed when a drop of 10 μM atrazine solution was directly applied to leaf tissue (data not shown).

### Patterns of singlet oxygen quenching mechanisms

Transcriptomic analysis showed that genes linked to the synthesis of ^1^O_2_-quenchers presented contrasted patterns of expression in relation to atrazine sensitivity and tolerance (Table 1). Some genes exhibited higher transcript levels under tolerance condition (SA) and repression under atrazine injury condition (MA), thus suggesting the possibility of more efficient quenching mechanisms in the presence of sucrose. Thus, seven genes encoding thioredoxin family proteins (At2g32920, At2g35010, At2g47470, At3g06730, At4g27080, At5g42980 and At5g60640) were characterized by significant atrazine repression of expression, which was lifted by sucrose-atrazine tolerance treatment (Table 1). Only two genes encoding thioredoxin family proteins exhibited higher expression under atrazine treatment (At5g06690 and At1g08570) than under sucrose plus atrazine treatment (Table 1). In contrast, two thioredoxin genes (At1g69880 and At1g45145) and one thioredoxin reductase gene (At2g17420) were significantly induced under tolerance conditions (SA) compared to atrazine treatment (MA) (Table 1). Thioredoxins have been shown to be involved in supplying reducing power to reductases detoxifying lipid hydroperoxides or repairing oxidized proteins [37]. Thioredoxins could also act as regulators of scavenging mechanisms [38-40] and as components of signalling pathways of plant antioxidant network. Finally, Das and Das [41] presented evidence that human thioredoxin was a powerful ^1^O_2 _quencher, which could protect cells and tissues against oxidative stress.

**Table 1:** Expression of genes involved in singlet oxygen quenching after 24 hours of treatment.

			Log_2_(ratio)		
			
Accession number	Gene description	Localisation	Treatment comparison		
			S/M	MA/M	SA/M
At1g08570	Thioredoxin family protein	No classification	nde	1.04	nde
At1g45145	Thioredoxin H-type 5 (TRX-H-5) (TOUL)	Cytosol	nde	nde	0.75
At1g69880	Thioredoxin, putative	No classification	2.05	nde	2.42
At2g17420	Thioredoxin reductase 2/NADPH-dependent thioredoxin reductase 2 (NTR2)	Cytoplasm	1.22	nde	1.51
At2g32920	Thioredoxin family protein	Endomembrane system	nde	-1.54	nde
At2g35010	Thioredoxin family protein	Mitochondrion	nde	-1.00	nde
At2g47470	Thioredoxin family protein	Endomembrane system	nde	-1.74	nde
At3g06730	Thioredoxin family protein	Chloroplast	nde	-0.74	nde
At4g27080	Thioredoxin family protein	Endoplasmic reticulum	nde	-0.96	nde
At5g06690	Thioredoxin family protein	Chloroplast	-1.15	1.14	nde
At5g42980	Thioredoxin H-type 3 (TRX-H-3) (GIF1)	Cytosol	nde	-0.94	nde
At5g60640	Thioredoxin family protein	Endomembrane system	nde	-1.19	nde
					
At1g06570	4-hydroxyphenylpyruvate dioxygenase (HPD)	Chloroplast	-0.75	3.18	2.11
At3g04870	Zeta-carotene desaturase (ZDS1)/carotene 7.8-desaturase	Chloroplast	nde	0.94	nde
At4g25700	Beta-carotene hydroxylase	Chloroplast	nde	1.07	nde
					
At1g08550	Violaxanthin de-epoxidase precursor. putative (AVDE1)	Photosystem II	-1.26	0.91	nde
					
At3g26900	Shikimate kinase family protein	Chloroplast	nde	1.69	nde
At4g36810	Geranylgeranyl pyrophosphate synthase (GGPS1)/GGPP synthetase/farnesyltranstransferase	Chloroplast	nde	0.88	nde
					
At3g55610	Delta 1-pyrroline-5-carboxylate synthetase B/P5CS B (P5CS2)	Cytoplasm	0.82	3.63	2.24

Relative expressions of gene are given with their log_2_(ratio) for sucrose versus mannitol (S/M), mannitol plus atrazine versus mannitol (MA/M) and sucrose plus atrazine versus mannitol (SA/M) comparison. nde: not differentially expressed. Genes with a Bonferroni *P*-value higher than 5% were considered as being not differentially expressed as described by Lurin et al. [85].

Another group of genes exhibited induction of expression under atrazine conditions, whereas they were less induced or not differentially expressed under sucrose-atrazine conditions. Activation of these genes might reflect stress signalling due to high ^1^O_2 _content in atrazine treated-cells, as revealed by ROS detection ((Fig. 1 and Additional file 1). Some of these genes belonged to carotenoid biosynthesis pathways, such as Zeta-carotene desaturase *ZDS1 *(At3g04870), beta-carotene hydroxylase (At4g25700) or 4-hydroxyphenylpyruvate dioxygenase *HPD *(At1g06570) (Table 1). Carotenoids, which are known to act in chloroplasts as accessory pigments in light harvesting, can detoxify ^1^O_2 _and triplet chlorophyll and dissipate excess excitation energy [9].

Transcriptome profiling was carried out after 24 hours of treatment [18]. Measurements of carotenoid levels at different times of treatment showed that modifications were most contrasted after 48 hours of treatment [18]. Thus, given the potential delay between transcription and metabolic fluxes, modifications of carotenoid levels after 48 hours of treatment were compared with transcript-level modifications after 24 hours of treatment. Carotenoid (xanthophylls and carotenes) levels in *Arabidopsis thaliana *plantlets after 48 hours of treatment are presented in Table 2. Atrazine treatment tended to reduce carotenoid contents, while addition of sucrose in presence of atrazine maintained carotenoid levels near control levels. However, carotenoid/chlorophyll ratios were not significantly different, thus indicating that the photoprotection role of carotenoids was maintained in the presence of atrazine.

**Table 2:** Carotenoid content and carotenoid/chlorophyll ratios in leaves of *Arabidopsis thaliana *plantlets after 48 hours of treatment.

Treatment	Carotenoid content (Mean ± SE) μg g^-1 ^FW	Carotenoid/Chlorophyll ratios
Mannitol (M)	78.6 ± 0.3	0.172 ± 0.008
Sucrose (S)	78.8 ± 0.6	0.168 ± 0.009
Mannitol atrazine (MA)	61.2 ± 0.6	0.176 ± 0.012
Sucrose atrazine (SA)	72.1± 0.8	0.186 ± 0.010

Higher induction by atrazine treatment was also found for the violaxanthin de-epoxidase precursor (At1g08550) gene, which is involved in the xanthophyll cycle (Table 1). Together with carotenoids, zeaxanthin, synthesized from violaxanthin *via *the xanthophyll cycle, protects chloroplasts by accepting excitation energy from singlet chlorophyll [42]. Two genes involved in the shikimate (shikimate kinase, At3g26900) and terpenoid pathways (geranylgeranyl pyrophosphate synthase, At4g36810), which are essential for tocopherol synthesis [43], were also induced by the herbicide and not differentially expressed by the tolerance treatment (SA) (Table 1). The antioxidant tocopherol is known to scavenge oxygen free radicals, lipid peroxy radicals and ^1^O_2 _[44]. Finally, the presence of atrazine alone was found to induce the At3g55610 gene, which is involved in proline synthesis, with a higher intensity than under conditions of combination with sucrose (Table 1). Proline is also known to be an ^1^O_2 _quencher [45].

### Patterns of superoxide radical scavenging mechanisms

Excess of superoxide radical caused by numerous environmental stresses is detoxified by superoxide dismutase (SOD) enzymes and converted into H_2_O_2_. Seven isoenzymes have been identified, differing by their metal cofactor (Fe, Mn, or Cu and Zn), in *Arabidopsis thaliana *[46]. Transcriptome profiling was carried out after 24 hours of treatment [18]. Measurements of enzyme activities at different times of treatment showed that modifications were most contrasted after 48 hours of treatment (data not shown). Thus, given the potential delay between transcription and protein synthesis, modifications of global SOD activities after 48 hours of treatment were compared with modifications of SOD-encoding transcript levels after 24 hours of treatment.

**Figure 4:**
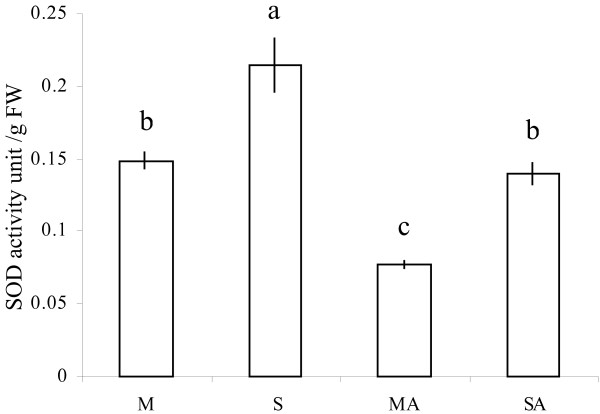
**Effects of atrazine and sucrose on SOD enzyme activity**. SOD activity was measured in protein extracts from 3-week-old MS-grown *Arabidopsis thaliana *plantlets subjected to subsequent treatment (48 hours) with 80 mM mannitol (M), 80 mM sucrose (S), 80 mM mannitol plus 10 μM atrazine (MA) or 80 mM sucrose plus 10 μM atrazine (SA). SOD activity is expressed in unit/g FW as defined in Methods. Statistical analysis was carried out as described in Methods.

SOD activity (Fig. 4) was decreased by atrazine treatment (MA) in comparison to the mannitol control (M). In contrast, addition of sucrose in the presence of atrazine (SA) maintained a functional level of SOD activity equivalent to that of the mannitol control. Since sucrose alone was found to increase SOD activity, it thus seemed that sucrose might balance the negative effect of atrazine in the situation of SA treatment.

Among the six isoenzyme-encoding genes represented in this microarray analysis (Table 3), three exhibited significant variations of transcript levels in comparison with control conditions, thus suggesting their potential involvement in O_2_^.-^-detoxifying processes in relation to atrazine sensitivity and tolerance. Three genes, encoding CSD1, MSD1, FSD3, were characterized by significant repression under conditions of atrazine treatment compared to control, in accordance with the measurement of global SOD activity (Fig. 4). The *CSD1 *gene (At1g08830), encoding cytosolic Cu-Zn superoxide dismutase, exhibited an induction under tolerance conditions (SA). In contrast, *MSD1 *(At3g10920) and *FSD3 *(At5g23310) genes, which, respectively, encode mitochondrial and chloroplastic superoxide dismutases, were not differentially expressed in the presence of sucrose. Exogenous sucrose, whether combined or not with atrazine, therefore re-established the basal level of transcripts (Table 3) and of global activity (Fig. 4), thus avoiding the repressive effects of the herbicide.

**Table 3:** Expression of genes encoding enzymes involved in O_2_^.- ^scavenging after 24 hours of treatment.

			Log_2_(ratio)		
			
Accession number	Gene description	Localisation	Treatment comparison		
			S/M	MA/M	SA/M
At1g08830	Superoxide dismutase (Cu-Zn) (SODCC)/copper/zinc superoxide dismutase (CSD1)	Cytoplasm	0.80	-0.70	1.22
At2g28190	Superoxide dismutase (Cu-Zn). chloroplast (SODCP)/copper/zinc superoxide dismutase (CSD2)	Chloroplast	-0.73	nde	-0.76
At3g10920	Superoxide dismutase (Mn). mitochondrial (SODA)/manganese superoxide dismutase (MSD1)	Mitochondrion	nde	-1.23	nde
At4g25100	Superoxide dismutase (Fe). chloroplast (SODB)/iron superoxide dismutase (FSD1)	Chloroplast	nde	nde	nde
At5g18100	Superoxide dismutase (Cu-Zn)/copper/zinc superoxide dismutase (CSD3)	Peroxisome	nde	nde	nde
At5g23310	Superoxide dismutase (Fe)/iron superoxide dismutase 3 (FSD3)	Chloroplast	nde	-1.34	nde

Relative expressions of gene are given with their log_2_(ratio) for sucrose versus mannitol (S/M), mannitol plus atrazine versus mannitol (MA/M) and sucrose plus atrazine versus mannitol (SA/M) comparison. nde: not differentially expressed. Genes with a Bonferroni *P*-value higher than 5% were considered as being not differentially expressed as described by Lurin et al. [85].

### Potential origin of hydrogen peroxide accumulation in the presence of atrazine

H_2_O_2 _contents in atrazine-treated plantlets in the presence or absence of sucrose seemed to be independent from O_2_^.- ^dismutation. Indeed, O_2_^.- ^level was low in sucrose plus atrazine-treated plantlets and null in atrazine-treated plantlets. Thus, atrazine, in the absence or presence of sucrose, may promote H_2_O_2_-producing pathways independently from O_2_^.- ^and ^1^O_2 _accumulation. Transcriptomic analysis revealed induction of two genes encoding H_2_O_2_-producing enzymes in atrazine-treated plantlets in the presence or absence of sucrose (SA and MA) (Table 4): amine oxidase (At1g57770) and proline oxidase (At3g30775). Moreover, other potentially H_2_O_2_-producing genes were upregulated either under MA condition: a glycolate oxidase putative gene (At3g14420) and a glyoxal oxidase-related gene (At3g53950); or under SA condition: two genes encoding acyl-CoA oxidases (At4g16760, At5g65110) (Table 4).

**Table 4:** Expression of genes potentially encoding H_2_O_2_-producing enzymes after 24 hours of treatment.

			Log_2_(ratio)		
			
Accession number	Gene description	Localisation	Treatment comparison		
			S/M	MA/M	SA/M
At1g57770	Amine oxidase family	Chloroplast	nde	1.59	0.80
At3g14420	(S)-2-hydroxy-acid oxidase, peroxisomal, putative/glycolate oxidase, putative/short chain alpha-hydroxy acid oxidase, putative Proline oxidase, mitochondrial/osmotic stress-	Peroxisome	-1.08	1.33	nde
At3g30775	responsive proline dehydrogenase (POX) (PRO1) (ERD5)	Mitochondrion	nde	2.51	1.22
At3g53950	Glyoxal oxidase-related	Endomembrane system	nde	1.00	nde
At4g16760	Acyl-CoA oxidase (ACX1)	Peroxisome	0,87	nde	1.48
At5g65110	Acyl-CoA oxidase (ACX2)	Peroxisome	0.91	nde	1.83

Relative expressions of gene are given with their log_2_(ratio) for sucrose versus mannitol (S/M), mannitol plus atrazine versus mannitol (MA/M) and sucrose plus atrazine versus mannitol (SA/M) comparison. nde: not differentially expressed. Genes with a Bonferroni *P*-value higher than 5% were considered as being not differentially expressed as described by Lurin et al. [85].

### Patterns of hydrogen peroxide scavenging mechanisms

In order to investigate the efficiency of hydrogen peroxide scavenging mechanisms, global H_2_O_2_-scavenging enzyme activities and transcript levels of related genes were analysed. As explained above, modifications of enzyme activities after 48 hours of treatment were compared with modifications of transcript levels after 24 hours of treatment.

H_2_O_2 _can be principally scavenged by two different ways: ascorbate-glutathione cycles and catalases, which play important roles in plant defence and senescence. Ascorbate-glutathione cycles are catalysed by a set of four enzymes: ascorbate peroxidase (APX), monodehydroascorbate reductase (MDAR), glutathione-dependent dehydroascorbate reductase (DHAR), and glutathione reductase (GR) [47].

**Figure 5:**
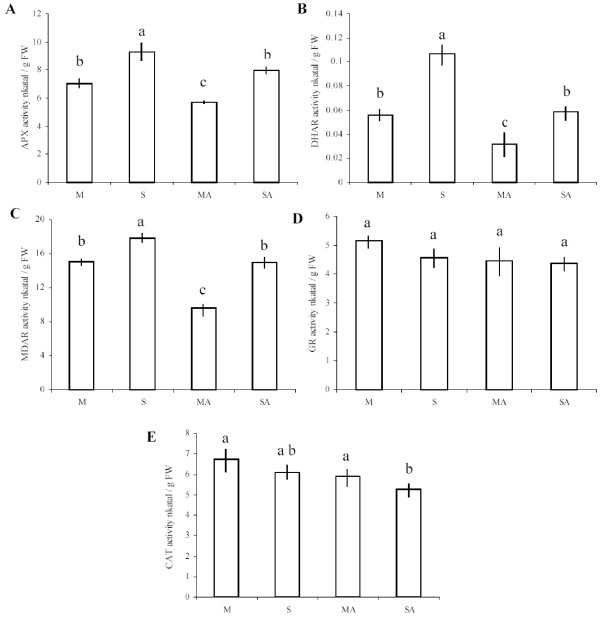
**Effects of atrazine and sucrose on antioxidative enzyme activities**. Activities of ascorbate peroxidase (APX) (A), dehydroascorbate reductase (DHAR) (B), monodehydroascorbate reductase (MDAR) (C), glutathione reductase (GR) (D) and catalase (CAT) (E) were measured in protein extracts from 3-week-old MS-grown *Arabidopsis thaliana *plantlets subjected to subsequent treatment (48 hours) with 80 mM mannitol (M), 80 mM sucrose (S), 80 mM mannitol plus 10 μM atrazine (MA) or 80 mM sucrose plus 10 μM atrazine (SA). Enzymatic activities are expressed in nkatal/g FW, nkatal corresponds to the amount of enzymatic activity that catalyzes the transformation of one nmole of substrate per second. Statistical analysis was carried out as described in Methods.

The five enzymes belonging to H_2_O_2_-scavenging mechanisms presented two different profiles of global activity according to the different treatments. The majority of enzymes involved in ascorbate-glutathione cycles (APX, DHAR and MDAR) were differentially affected by the different treatments. Activity of these three enzymes was significantly reduced by addition of atrazine, while sucrose treatment had an opposite effect and significantly increased these activities (Fig. 5a, b, c). The tolerance condition (SA) succeeded to limit repressive effects of the herbicide and maintained enzyme activities at the control level. The fourth enzyme of the ascorbate-glutathione cycles, GR, did not present any significant variation of activity between the different treatments (Fig. 5d). Finally, catalase exhibited slightly lower activity under conditions of sucrose plus atrazine, when compared to control and atrazine-containing medium (Fig. 5e).

The repressive effect of atrazine in the absence of sucrose (MA treatment) on APX global activity was correlated with a general repression of *APX *genes (Fig. 5a, Table 5). Among the six *APX *genes present in the microarray, the cytosolic *APX1 *(At1g07890), the stromal *sAPX *(At4g08390) and the chloroplastic *APX4 *(At4g09010) genes exhibited important decrease of transcript levels under conditions of atrazine treatment (MA) compared to mannitol control, while the other *APX *genes were not differentially expressed in the presence of atrazine. Whereas *APX4 *expression remained downregulated in the presence of sucrose plus atrazine, this tolerant condition balanced the repressive effects of atrazine for *APX1 *and *sAPX *genes, which recovered a level of transcript similar to the control. Finally, and in contrast with global APX activity, the thylakoid-bound *tAPX *(At1g77490) gene was not affected by atrazine, while sucrose repressed its expression under S and SA conditions.

**Table 5:** Expression of genes encoding enzymes involved in ascorbate-glutathione cycles after 24 hours of treatment.

			Log_2_(ratio)		
			
Accession number	Gene description	Localisation	Treatment Comparison		
			S/M	MA/M	SA/M
At1g07890	L-ascorbate peroxidase 1. cytosolic (APX1)	Cytosol	nde	-1.92	nde
At1g77490	L-ascorbate peroxidase. thylakoid-bound (tAPX)	Chloroplast	-1.03	nde	-1.08
At3g09640	L-ascorbate peroxidase 2 (APX2)	Cytoplasm	nde	nde	nde
At4g08390	L-ascorbate peroxidase. stromal (sAPX)	Chloroplast	1.46	-1.18	nde
At4g09010	L-ascorbate peroxidase 4 (APX4)	Chloroplast	-0.79	-1.12	-1.40
At4g35000	L-ascorbate peroxidase 3 (APX3)	Peroxisome	nde	nde	nde
					
At1g75270	Dehydroascorbate reductase (DHAR2)	Cytoplasm	1.92	-0.91	2.70
At5g16710	Dehydroascorbate reductase (DHAR3)	Chloroplast	nde	nde	nde
At5g36270	Dehydroascorbate reductase. putative	Cytoplasm	0.80	nde	1.20
					
At1g63940	Monodehydroascorbate reductase (MDAR5)	Chloroplast	nde	nde	nde
At3g09940	Monodehydroascorbate reductase (MDAR3)	Cytoplasm	nde	nde	nde
At3g27820	Monodehydroascorbate reductase (MDAR4)	Cytoplasm	nde	nde	nde
At3g52880	Monodehydroascorbate reductase (MDAR1)	Cytoplasm	nde	nde	nde
At5g03630	Monodehydroascorbate reductase (MDAR2)	Cytoplasm	nde	-1.35	1.11
					
At3g24170	Glutathione reductase. putative (GR1)	Cytoplasm	1.15	nde	0.92
At3g54660	Gluthatione reductase. chloroplast (GR2)	Chloroplast	nde	nde	nde

Relative expressions of gene are given with their log_2_(ratio) for sucrose versus mannitol (S/M), mannitol plus atrazine versus mannitol (MA/M) and sucrose plus atrazine versus mannitol (SA/M) comparison. nde: not differentially expressed. Genes with a Bonferroni *P*-value higher than 5% were considered as being not differentially expressed as described by Lurin et al. [85].

Dehydroascorbate reductase (DHAR) is a key component of the ascorbate recycling system. DHAR recycles dehydroascorbate into ascorbate by using reduced glutathione as a reductant. Two functional *DHAR *genes, among three that are encoded in the *Arabidopsis thaliana *genome, plus a putative gene, were represented in the microarray (Table 5). The cytosolic *DHAR2 *(At1g75270) and putative *DHAR *(At5g36270) genes exhibited high induction in the combined presence of sucrose and atrazine, and, respectively, a slight repression or no variation in the presence of atrazine in comparison to control condition. In contrast to the repressive effects of atrazine, which were associated with a decrease of DHAR activity, the increase of *DHAR *transcript levels in the combined presence of sucrose and atrazine was not associated with an increase of global DHAR enzyme activity (Fig. 5b).

Reduction of monodehydroascorbate by monodehydroascorbate reductase (MDAR) is also an important step in ascorbate recycling. Among the five *MDAR *genes present in the microarray, only cytosolic *MDAR2 *(At5g03630) exhibited differential expression patterns according to the treatment applied. While atrazine repressed its expression, the protective combination of sucrose and atrazine upregulated it (Table 5). Atrazine was also found to decrease global MDAR activity, while sucrose plus atrazine treatment resulted in maintenance of MDAR activity relatively to the mannitol control (Fig. 5c).

Glutathione serves as a reductant in oxidation-reduction processes, such as recycling of oxidised ascorbate by dehydroascorbate reductase [48]. Reduction of oxidised glutathione is catalysed by glutathione reductase (GR), which requires NADPH. Among the two isoenzymes present in the microarray, only the cytosolic glutathione reductase *GR1 *(At3g24170) was found to be induced by sucrose-atrazine and sucrose treatments, while no variation of expression was detected in the presence of atrazine (Table 5). These variations of expression were not associated with changes of global GR activity, since no significant difference of activity was observed between treatments (Fig. 5d).

The second way to reduce H_2_O_2 _content in cells is activation of catalases (CAT), which catalyse dismutation of H_2_O_2 _into water and oxygen [49]. Little variation of transcript levels was detected for the three catalase isoenzymes (Table 6). *CAT2 *(At4g35099) exhibited upregulation by atrazine stress, while *CAT3 *was slightly downregulated by the protective sucrose plus atrazine treatment. In relation with these slight changes of transcript levels (Table 6), global catalase activities were found to show little variation (Fig. 5e).

**Table 6:** Expression of genes encoding enzymes involved in H_2_O_2 _scavenging after 24 hours of treatment.

			Log_2_(ratio)		
			
Accession number	Gene description	Localisation	Treatment comparison		
			S/M	MA/M	SA/M
At1g20620	Catalase 3	Peroxisome	nde	nde	-0.74
At1g20630	Catalase 1	Peroxisome	nde	nde	nde
At4g35090	Catalase 2	Peroxisome	nde	0.96	nde

Relative expressions of gene are given with their log_2_(ratio) for sucrose versus mannitol (S/M), mannitol plus atrazine versus mannitol (MA/M) and sucrose plus atrazine versus mannitol (SA/M) comparison. nde: not differentially expressed. Genes with a Bonferroni *P*-value higher than 5% were considered as being not differentially expressed as described by Lurin et al. [85].

## Discussion

### Characterisation of the impact of atrazine on ROS patterns

ROS patterns appear to depend strongly on the nature and intensity of stress conditions applied to plants [50]. It is therefore of great importance to characterise ROS accumulation kinetics associated with a particular stress, and not to rely on expected effects. Thus, while, as expected, atrazine inhibition of photosystem II was associated with ^1^O_2 _accumulation [7] (Fig. 1 and Additional file 1), decrease of superoxide radical levels and increase of H_2_O_2 _levels were also observed (Figs. 2, 3 and Additional files 2, 3). This disagreed with the proposed, but experimentally unproven, accumulation of superoxide radical by triazine treatment in Arabidopsis leaves [51]. It was however coherent with inhibition of photosynthetic activity and of the Mehler reaction, whereby superoxide radical is formed by reduction of oxygen at the PSI site [52]. Atrazine binding to D1 protein of PSII and inhibition of electron feeding to PSI were indeed likely to decrease superoxide radical production by blocking the Mehler reaction.

The induction of H_2_O_2 _accumulation by atrazine was all the more surprising as it occurred rapidly after transfer to atrazine (Fig. 3 and Additional file 3) and in the absence or in the presence of sucrose, which by itself had a negative effect on H_2_O_2 _accumulation. This is, to our knowledge, the first demonstration of rapid *in vivo *H_2_O_2 _accumulation under conditions of atrazine treatment. The negative effect of sucrose on H_2_O_2 _accumulation was consistent with the previously-described repression of protein and lipid catabolism, including a number of oxidase-based processes, by soluble sugars [18,53]. In contrast, atrazine by itself was found to induce a number of genes encoding oxidases, the most highly induced being a gene encoding a proline oxidase (Table 4). Since this induction occurred prior to significant impairment of photosystems and phototrophic growth [18], it could not be ascribed to a situation of metabolic starvation. Activation of protein and lipid catabolism and of oxidase-based processes has been reported to occur under conditions of carbohydrate limitation or starvation [54,55]. In this context, it was extremely interesting that, in the presence of exogenous sucrose, i.e. in a situation of carbohydrate optimum, atrazine was able to induce a number of oxidase-encoding genes and other genes typical of carbohydrate-limitation response, such as the gene encoding isovaleryl-CoA dehydrogenase [18,56].

Numerous abiotic stressors, including xenobiotics, are known to produce oxidative stress in photosynthetic organisms. This is the case for benzoxazolinone [57], metronidazole, dinoterb [58], acetochlor [59], copper [60], wounding [61] and high light [6]. Studies on these stresses mainly focus on the effects of a single ROS and rarely consider the effects of ROS combination. However, ROS are chemically distinct and selectively perceived for the fine control of adjusting antioxidants and photosynthesis to different environmental stress conditions [62]. Indeed, cross-talk between ^1^O_2 _and H_2_O_2 _has been clearly demonstrated by Laloi et al. [50], who suggested antagonistic interactions between ^1^O_2 _and H_2_O_2 _with a reduction of ^1^O_2_-mediated cell death and stress signalling response by H_2_O_2 _content. In contrast, the present condition of atrazine treatment, which eventually leads to plantlet death, was characterised by high ^1^O_2_, high H_2_O_2 _and low superoxide radical levels. Laloi et al. [63], who described antagonistic effects between ^1^O_2_, and H_2_O_2_, modulated H_2_O_2 _levels in Arabidopsis transgenic plants at the plastid level. It was thus possible that non-antagonistic effects of H_2_O_2 _and ^1^O_2 _under conditions of atrazine treatment were due to differences of ROS localisation.

Finally, among the set of 29 induced transcription factors that have been characterized as ^1^O_2_-specific by Gadjev et al. [64], only one was slightly induced (data not shown) during the course of atrazine treatment despite the high accumulation of singlet oxygen (Fig. 1 and Additional file 1). The analysis of ^1^O_2 _responses by Gadjev et al. [64] was based on studies of the Arabidopsis conditional *flu *mutant [65]. It was thus clear that other signals than ^1^O_2 _were perceived by atrazine-treated plantlets or that atrazine-induced ^1^O_2 _accumulation involved other processes and responses than *flu*-mutant-dependent ^1^O_2 _accumulation [50,64,65]. However, full characterisation of the signalling events associated with xenobiotic exposure in plants remains to be carried out.

### Impairment of antioxidant defences in the presence of atrazine

Atrazine-treated plantlets were characterised by low O_2_^.- ^levels and high H_2_O_2 _levels, in contrast with sucrose-treated atrazine-tolerant plants, which showed high O_2_^.- ^and high H_2_O_2 _levels. These differences of ROS patterns were associated with striking differences of gene expressions and enzyme activities involved in ROS-scavenging pathways.

Thus, atrazine sensitivity was associated with down-regulation of key players of H_2_O_2 _scavenging. Among the four enzymes involved in ascorbate-glutathione cycles, which are essential to remove large amounts of H_2_O_2 _generated by stress [48,66], three enzymes (APX, MDAR and DHAR) exhibited a significant decrease of global activities in atrazine-treated plantlets. Moreover, this repression was correlated with a global down-regulation of typical corresponding transcripts (*APX1, sAPX, DHAR2*, and *MDAR2*), which, conversely, have already been shown to undergo important induction during responses to several environmental abiotic stresses. APX1, a cytosolic enzyme, has previously been described as a central component of the reactive oxygen gene network of Arabidopsis [67]. Involvement of sAPX in response to oxidative stress has also been reported by transcriptional induction in the presence of H_2_O_2_, methylviologen, FeCl_3 _or UV treatments in soybean seedlings [68]. Finally, Yoshida et al. [69] reported the importance of DHAR2 under conditions of ozone treatment, with higher sensitivity to ozone in a *DHAR2*-deficient mutant, probably due to insufficient recycling of ascorbate.

Consequently, repression of these transcripts and decrease of the corresponding enzyme activities in the presence of atrazine might accentuate the effects of H_2_O_2 _accumulation by reduction of ascorbate recycling, thus leading to disruption of antioxidant mechanisms and propagation of atrazine injuries. It was thus clear that the effects of atrazine at transcript level [18] had actual negative consequences on biochemical defences and could be involved in xenobiotic sensitivity. This is strong evidence that xenobiotic sensitivity may be linked to gene regulation effects in plants. Correlatively, the situation of sucrose-induced tolerance was characterised by the lifting of atrazine repression, in the case of *APX1 *and *sAPX*, or by the induction by sucrose-atrazine combination, in the case of *DHAR2 *and *MDAR2*. These positive effects on transcript levels were associated with maintenance of the corresponding enzyme activities at control levels. Although ROS can mediate induction of protective proteins involved in the stability of specific mRNAs [70], they can also cause RNA oxidative damages and induce protein inactivation and degradation [71]. Increase of transcript levels was therefore likely to be an adaptive response to ensure protein synthesis under stress conditions resulting in higher protein turnover.

The decline of O_2_^.- ^levels in atrazine-treated plantlets, which, as explained above, could be ascribed to inhibition of electron transfer through PSI, was associated with a general repression of transcripts encoding the different isoenzymes of SOD and with a decrease of the global activity of this O_2_^.-^-scavenging enzyme family, thus indicating that atrazine-treated cells responded to the low superoxide radical situation. Association of low O_2_^.- ^and high H_2_O_2 _may be a cause for the ill-adapted response of anti-oxidant defences in atrazine-treated plantlets, thus suggesting that further work should be carried out on the adaptation of organisms to fluctuations of ROS combinations.

### Mechanisms of sucrose-induced tolerance to singlet oxygen

In contrast with non-induction of H_2_O_2_-scavenging systems, atrazine-treated plantlets seemed to be able to sense the increase of ^1^O_2 _levels and induce some genes potentially involved in ^1^O_2 _quenching (Table 1). Thus, atrazine-treated plantlets, in the absence or presence of sucrose showed increased expression of 4-Hydroxyphenylpyruvate dioxygenase (HPD) gene (At1g06570), which could be involved in the maintenance of the photoprotective role of carotenoids. The At5g06690 gene, encoding a chloroplastic thioredoxin, which is a potential ^1^O_2_-quencher [41], was also induced in atrazine-treated plantlet. However, generally, the thioredoxin gene family was negatively affected by atrazine treatment, with 7 genes among 12 significantly repressed by atrazine. Correlatively, ten of these twelve genes showed lifting of repression or significant induction in the combined presence of sucrose and atrazine. Nevertheless, most of these genes encoded extraplastidial thioredoxins or thioredoxins of unknown localisation (Table 1). The link of thioredoxin gene family differential expression with efficient ^1^O_2 _quenching in the presence of atrazine plus sucrose (Fig. 1 and Additional file 1) was thus difficult to ascertain. On one hand, several studies have shown the efficiency of thioredoxins in maintenance of cellular reductant environment and in cytoprotective mechanisms [37-40]. On the other hand, efficient ^1^O_2 _quenching in the case of PSII inhibition by atrazine would require the involvement of chloroplastic TRXs. Two TRX genes, At3g06730 and At5g06690, have been described as encoding chloroplastic TRXs (Table 1). Expression of these two genes showed contrasted patterns in the presence of atrazine or in the presence of sucrose plus atrazine, with At3g06730 being repressed by atrazine, and At5g06690 being induced by atrazine, whereas the presence of sucrose and atrazine resulted in a return to baseline levels. Thus, further work would be required to analyse the physiological significance of this different pattern, and whether the At3g06730 gene product may play an important role in atrazine responses. Further work would also be required to characterise the potential importance of At1g69880 and At2g17420 TRX genes, which are induced by sucrose and by sucrose plus atrazine, in sucrose-induced tolerance.

## Conclusion

Parallel and integrative analysis therefore revealed correlated modifications of ROS patterns, antioxidant biochemical defences, and corresponding transcript markers, under conditions of atrazine sensitivity and of sucrose-induced tolerance. Atrazine injury was shown to be related with increased levels of singlet oxygen and hydrogen peroxide in leaves. Sucrose-treated plantlets were able to sense changing ROS levels and activate efficient quenching and antioxidant systems, whereas, in the absence of sucrose protection, atrazine-treated plantlets failed to develop fully these defence mechanisms. It thus seemed that atrazine may generate signals that activate some H_2_O_2_-producing pathways, and that impair the induction and activation of antioxidant defence mechanisms. Further work is needed to characterise completely the complex signalling events associated with xenobiotic exposure in plants.

## Methods

### Plant material and growth conditions

Seeds of *Arabidopsis thaliana *(ecotype Colombia, Col0) were surfaced-sterilized in bayrochlore/ethanol (1/1, v/v), rinsed in absolute ethanol and dried overnight. Germination and growth were carried out under axenic conditions in square Petri dishes. After seeds were sowed, Petri dishes were placed at 4°C for 48 h in order to break dormancy and homogenize germination and transferred to a control growth chamber at 22°C under a 16 h light period regime at 85 μmol m^-2 ^s^-1 ^for 3 weeks. Growth medium consisted of 0.8% (w/v) agar in 1× Murashige and Skoog (MS) basal salt mix (M5519, Sigma-Aldrich) adjusted to pH 5.7. Plantlets were then transferred to fresh MS agar medium containing 80 mM mannitol (M, control), 80 mM mannitol and 10 μM atrazine (MA, lethal treatment), 80 mM sucrose (S, sugar treatment) and 80 mM sucrose and 10 μM atrazine (SA, tolerance treatment).

### Chlorophyll and carotenoid extraction and quantification

Pigments were extracted by pounding aerial parts of seedlings in 80% acetone, and absorbance of the resulting extracts was measured at 663 nm, 646 nm and 470 nm. Levels of chlorophyll and total carotenoids (xanthophylls and carotenes) were determined from the equations given by Lichtenthaler and Wellburn [72]. Measurements were done on 3 replicas of 5–10 pooled seedlings each.

### Singlet oxygen staining

Three week-old plantlets were transferred for 12, 24, 48 or 72 hours to the different control and treatment media described above (M, S, MA and SA). Plantlets, prior to the transfer and at the end of the treatment, were immersed and infiltrated in the dark under vacuum with a solution of 100 μM Singlet Oxygen Sensor Green^® ^reagent (SOSG) (S36002, Invitrogen) [31] in 50 mM phosphate potassium buffer (pH 7.5). Infiltrated plantlets were then placed again on control and treatment media during 30 minutes in the light before being photographed under the microscope. Following excitation at 480 nm, the fluorescence emission at 530 nm was then detected by an Olympus BX41 spectrofluorometer coupled with a camera. The presence of red chlorophyll autofluorescence from chloroplasts did not alter the green fluorescence of SOSG. The infiltration method was chosen in order to measure singlet oxygen levels after the different times of treatment. Image analysis and quantification of level fluorescence were performed using the ImageJ software [73]. Experiments were repeated four times on at least 15 plantlets.

### Superoxide radical staining

The nitroblue tetrazolium (NBT) (N6876, Sigma-Aldrich) staining method of Rao and Davis [74] was modified as follows for *in situ *detection of superoxide radical. Three week-old plantlets were transferred for 12, 24, 48 or 72 hours to the different control and treatment media described above (M, S, MA and SA). Plantlets, prior to the transfer and at the end of the treatment, were immersed and infiltrated under vacuum with 3.5 mg ml^-1 ^NBT staining solution in potassium phosphate buffer (10 mM) containing 10 mM NaN_3_. After infiltration, stained plantlets were bleached in acetic acid-glycerol-ethanol (1/1/3) (v/v/v) solution at 100°C during 5 min. Plantlets were then stored in a glycerol-ethanol (1/4) (v/v) solution until photographs were taken. O_2_^.- ^was visualized as a blue color produced by NBT precipitation. A modified version of previously described assays for superoxide quantification was used [75,76]. Briefly, NBT-stained plantlets were ground in liquid nitrogen, the formazan content of the obtained powder was solubilized in 2 M KOH-DMSO (1/1.16) (v/v), and then centrifuged for 10 min at 12,000 *g*. The A_630 _was immediately measured, and compared with a standard curve obtained from known amounts of NBT in the KOH-DMSO mix. Experiments were repeated four times on at least 15 plantlets.

### Hydrogen peroxide staining

The H_2_O_2 _staining agent, 3,3'diaminobenzidine (DAB) (D5637, Sigma-Aldrich), was dissolved in H_2_O and adjusted to pH 3.8 with KOH. The DAB solution was freshly prepared in order to avoid auto-oxidation [32]. Three week-old plantlets were transferred for 12, 24, 48 or 72 hours to the different control and treatment media described above (M, S, MA and SA). Plantlets, prior to the transfer and at the end of the treatment, were immersed and infiltrated under vacuum with 1.25 mg ml^-1 ^DAB staining solution. Stained plantlets were then bleached in acetic acid-glycerol-ethanol (1/1/3) (v/v/v) solution at 100°C during 5 min, and then stored in glycerol-ethanol (1/4) (v/v) solution until photographs were taken. H_2_O_2 _was visualized as a brown color due to DAB polymerization. Quantification of H_2_O_2 _contents was determined using the method of Kotchoni et al. (2006) [77]. The DAB-stained plantlets were ground in liquid nitrogen. The resulting powder was homogenized in 0.2 M HClO_4_, and then centrifuged for 10 min at 12,000 *g*. The A_450 _was immediately measured and compared with a standard curve containing known amounts of H_2_O_2 _in 0.2 M HClO_4_-DAB. Experiments were repeated four times on at least 15 plantlets. The specificity of DAB staining towards H_2_O_2 _was assessed in control infiltrations in the presence of 10 mM ascorbic acid.

### Enzyme activities

Three week-old plantlets were transferred for 48 hours to the different control and treatment media described above (M, S, MA and SA). Whole plantlets (100 mg FW) were ground in liquid nitrogen to extract total proteins. The powder obtained was suspended in 500 μl of extraction buffer containing 50 mM phosphate buffer (pH 7.5), 1% (w/v) polyvinylpyrrolidone (PVP), 0.5% (v/v) Triton X-100, 1 mM EDTA and a cocktail of protease inhibitors (P9599, Sigma-Aldrich). In the specific case of APX activity measurement, the plant powder was suspended in 50 mM Hepes (pH 7) buffer containing 0.5 mM ascorbate, 0.5% (v/v) Triton X-100 and 1% (w/v) PVP. After centrifugation (15 min, 10,000 *g*), the supernatant was recovered and a second extraction of the pellet was identically realized. The two supernatants were pooled and constituted the total protein extract that was immediately used for enzyme activity measurement.

Superoxide dismutase (SOD) activity (EC 1.15.1.1) was determined using the method of Beauchamp and Fridovich [78] that spectrophotometrically measures inhibition of the photochemical reduction of nitroblue tetrazolium (NBT) at 560 nm. One unit of SOD activity was defined as the amount of enzyme required to inhibit the reduction rate of NBT by 50%. The reaction mixture contained 50 mM potassium phosphate buffer (pH 7.5), 10 mM methionine, 2 μM riboflavin, 0.1 mM EDTA, 70 μM NBT and enzyme sample. Reactions were carried out at 25°C under a light intensity of about 120 μmol m^-2 ^s^-1 ^for 10 min.

Ascorbate peroxidase (APX) activity (EC 1.11.1.11) was measured according to Nakano and Asada [79] by monitoring the rate of hydrogen peroxide-dependent oxidation of ascorbate at 290 nm (*E *= 2.8 mM^-1 ^cm^-1^). The reaction mixture contained 50 mM potassium phosphate buffer (pH 7), 0.5 mM ascorbic acid, 0.1 mM H_2_O_2_, 1 mM EDTA and enzyme sample.

Dehydroascorbate reductase (DHAR) activity (EC 1.8.5.1) was measured as described by Hossain and Asada [80]. DHAR was assayed spectrophotometrically by monitoring the increase in absorbance at 265 nm due to ascorbate formation (*E *= 14 mM^-1 ^cm^-1^). The reaction mixture, freshly prepared in N_2_-saturated buffer, consisted of 50 mM potassium phosphate buffer (pH 7), 0.5 mM dehydroascorbate, 5 mM reduced glutathione, 1 mM EDTA and enzyme sample. Correction was made for non-enzymatic reduction rate of DHA in absence of protein extract.

Monodehydroascorbate reductase (MDAR) activity (EC 1.6.5.4) was measured as described by Hossain et al. [81]. MDAR was assayed spectrophotometrically by following the decrease in absorbance at 340 nm due to NADH oxidation (*E *= 6.2 mM^-1 ^cm^-1^). The reaction mixture consisted of 50 mM buffer TES (pH 7.5), 0.1 mM NADH, 2.5 mM ascorbate, ascorbate oxidase (1 U ml^-1^) (Curcubita enzyme (EC 1.10.3.3), A0157, Sigma-Aldrich) and enzyme sample.

Glutathione reductase (GR) activity (EC 1.6.4.2) was measured as described by Smith et al. [82] following spectrophotometrically the disappearance of NADPH at 340 nm (*E *= 6.2 mM^-1 ^cm^-1^). The reaction mixture contained 50 mM Hepes-NaOH buffer (pH 7.5), 0.5 mM oxidized glutathione, 0.25 mM NADPH, 0.5 mM EDTA and enzyme sample.

Catalase (CAT) activity (EC 1.11.1.6) was measured spectrophotometrically at 250 nm by following the disappearance of H_2_O_2 _(*E *= 39.4 mM^-1 ^cm^-1^) in a reaction mixture containing 50 mM potassium phosphate buffer (pH 7) and protein extract. The reaction of dismutation was initiated by the addition of H_2_O_2 _(10 mM) as described by Aebi [83].

### Transcriptome profiling

Gene expression data were extracted from the transcriptomic profiling experiment registered as E-MEXP-411 in ArrayExpress [18,84]. Genes with a Bonferroni *P*-value higher than 5% were considered as being not differentially expressed as described by Lurin et al. [85]. Differentially expressed genes are those genes showing at least one *P*-value ≤ 0.05 after Bonferroni correction, in one of the MA/M, SA/M or S/M comparisons [18]. This *P*-value corresponds to genes whose Log_2_(ratio) was greater than 0.73 or lower than -0.73 (corresponding to 1.6586-fold changes). This transcriptomic experiment compared the RNA profiles of three-week-old MS-grown plantlets transferred for 24 hours to the different control and treatment media described above (M, S, MA and SA).

### Statistical analysis

Statistical analysis was carried out with the Minitab^® ^15.1.1.0 software (Minitab SARL, Paris, France). The non-parametrical Mann-Whitney test was used for the different comparisons of means. Means that were not significantly different (*P *> 0.05) show the same letter in graph representations.

## Abbreviations

APX: ascorbate peroxidase; CAT: catalase; DAB: diaminobenzidine; DHAR: dehydroascorbate reductase; GR: glutathione reductase; H_2_O_2_: hydrogen peroxide; HO^.^: hydroxyl radical; MDAR: monodehydroascorbate reductase; MS: Murashige and Skoog; NBT: nitroblue tetrazolium; O_2_: molecular oxygen; ^1^O_2_: singlet oxygen; O_2_^.-^: superoxide radical; PSII: photosystem II; ROS: reactive oxygen species; SOD: superoxide dismutase; SOSG: Singlet Oxygen Sensor Green^®^; DW: dry weight.

## Authors' contributions

FR, CS, MB, IC and GG conceived the study and designed experiments. FR, MB and GG performed the experiments. FR, CS, MB, IC and GG carried out analysis and interpretation of experimental data including statistical analyses. FR, CS, IC and GG wrote the manuscript. All authors read and approved the final manuscript.

## Supplementary Material

Additional file 1 **Singlet oxygen detections using the SOSG probe have been done on 3-week-old MS-grown *Arabidopsis thaliana *plantlets subjected to subsequent treatment (12, 24, 48 or 72 hours) with 80 mM mannitol (M), 80 mM sucrose (S), 80 mM mannitol plus 10 μM atrazine (MA) or 80 mM sucrose plus 10 μM atrazine (SA). Image analysis and quantification of fluorescence was performed using ImageJ software.** Changes in average intensities are shown as percentage of mean fluorescence intensity of MS-grown plantlets as control. Click here for file

Additional file 2 **Detections and quantification have been done on 3-week-old MS-grown *Arabidopsis thaliana *plantlets subjected to subsequent treatment (12, 24, 48 or 72 hours) with 80 mM mannitol (M), 80 mM sucrose (S), 80 mM mannitol plus 10 μM atrazine (MA) or 80 mM sucrose plus 10 μM atrazine (SA). Superoxide radical content was expressed as nmoles of reduced NBT per g DW.** Click here for file

Additional file 3 **Detections and quantification have been done on 3-week-old MS-grown *Arabidopsis thaliana *plantlets subjected to subsequent treatment (12, 24, 48 or 72 hours) with 80 mM mannitol (M), 80 mM sucrose (S), 80 mM mannitol plus 10 μM atrazine (MA) or 80 mM sucrose plus 10 μM atrazine (SA). Hydrogen peroxide content was expressed as μ moles of H_2_O_2 _per g DW.** Click here for file

## References

[B1] Salin ML (1991). Chloroplast and mitochondrial mechanisms for protection against oxygen toxicity. Free Radic Res Commun.

[B2] Mittler R, Vanderauwera S, Gollery M, Van Breusegem F (2004). Reactive oxygen gene network of plants. Trends in Plant Science.

[B3] Dat J, Vandenabeele S, Vranova E, Van Montagu M, Inze D, Van Breusegem F (2000). Dual action of the active oxygen species during plant stress responses. Cell Mol Life Sci.

[B4] Foyer CH, Noctor G (1999). Leaves in the dark see the light. Science.

[B5] Dalton TD, Shertzer HG, Puga A (1999). Regulation of gene expression by reactive oxygen. Annu Rev Pharmacol Toxicol.

[B6] Scandalios JG (2002). Oxidative stress responses – what have genome-scale studies taught us?. Genome Biol.

[B7] Rutherford AW, Krieger-Liszkay A (2001). Herbicide-induced oxidative stress in photosystem II. Trends in Biochem Sci.

[B8] Macpherson AN, Telfer A, Barber J, Truscott TG (1993). Direct-detection of singlet oxygen from isolated photosystem-II reaction centers. Biochim Biophys Acta.

[B9] Telfer A, Dhami S, Bishop SM, Phillips D, Barber J (1994). β-carotene quenches singlet oxygen formed by isolated photosystem-II reaction centers. Biochemistry.

[B10] Solomon KR, Baker DB, Richards RP, Dixon DR, Klaine SJ, LaPoint TW, Kendall RJ, Weisskopf CP, Giddings JM, Giesy JP (1996). Ecological risk assessment of atrazine in North American surface waters. Environ Toxicol Chem.

[B11] Clark GM, Goolsby DA, Battaglin WA (1999). Seasonal and annual load of herbicides from the Mississippi River basin to the Gulf of Mexico. Environ Sci Technol.

[B12] Millie DF, Hersh CM (1987). Statistical characterizations of the atrazine-induced photosynthetic inhibition of *Cyclotella meneghiniana *(Bacillariophyta). Aquat Toxicol.

[B13] Hersh CM, Crumpton WG (1989). Atrazine tolerance of algae isolated from 2 agricultural streams. Environ Toxicol Chem.

[B14] Sibony M, Rubin B (2003). Molecular basis for multiple resistance to acetolactate synthase-inhibiting herbicides and atrazine in *Amaranthus blitoides *(prostrate pigweed). Planta.

[B15] Sulmon C, Gouesbet G, Binet F, Martin-Laurent F, El Amrani A, Couee I (2007). Sucrose amendment enhances phytoaccumulation of the herbicide atrazine in *Arabidopsis thaliana*. Environ Pollut.

[B16] Sulmon C, Gouesbet G, El Amrani A, Couee I (2006). Sugar-induced tolerance to the herbicide atrazine in Arabidopsis seedlings involves activation of oxidative and xenobiotic stress responses. Plant Cell Rep.

[B17] Sulmon C, Gouesbet G, Couee I, El Amrani A (2004). Sugar-induced tolerance to atrazine in Arabidopsis seedlings: interacting effects of atrazine and soluble sugars on *psbA *mRNA and D1 protein levels. Plant Sci.

[B18] Ramel F, Sulmon C, Cabello-Hurtado F, Taconnat L, Martin-Magniette ML, Renou JP, Elamrani A, Couee I, Gouesbet G (2007). Genome-wide interacting effects of sucrose and herbicide-mediated stress in *Arabidopsis thaliana *: novel insights into atrazine toxicity and sucrose-induced tolerance. BMC Genomics.

[B19] Foyer CH (2001). Prospects for enhancement of the soluble antioxidants, ascorbate and glutathione. Biofactors.

[B20] DellaPenna D, Pogson BJ (2006). Vitamin synthesis in plants: Tocopherols and carotenoids. Annu Rev Plant Biol.

[B21] Asada K (2006). Production and scavenging of reactive oxygen species in chloroplasts and their functions. Plant Physiol.

[B22] Bowler C, Slooten L, Vandenbranden S, De Rycke R, Botterman J, Sybesma C, Van Montagu M, Inze D (1991). Manganese superoxide dismutase can reduce cellular damage mediated by oxygen radicals in transgenic plants. EMBO J.

[B23] Bowler C, Alliotte T, De Loose M, Van Montagu M, Inze D (1989). The induction of manganese superoxide dismutase in response to stress in *Nicotiana plumbaginifolia*. EMBO J.

[B24] Perl A, Perltreves R, Galili S, Aviv D, Shalgi E, Malkin S, Galun E (1993). Enhanced oxidative-stress defense in transgenic potato expressing tomato Cu, Zn superoxide dismutases. Theor Appl Genet.

[B25] Gupta AS, Heinen JL, Holaday AS, Burke JJ, Allen RD (1993). Increased resistance to oxidative stress in transgenic plants that overexpress chloroplastic Cu/Zn superoxide-dismutase. Proc Natl Acad Sci USA.

[B26] Martin T, Oswald O, Graham IA (2002). Arabidopsis seedling growth, storage lipid mobilization, and photosynthetic gene expression are regulated by carbon: nitrogen availability. Plant Physiol.

[B27] Hideg E, Barta C, Kalai T, Vass I, Hideg K, Asada K (2002). Detection of singlet oxygen and superoxide with fluorescent sensors in leaves under stress by photoinhibition or UV radiation. Plant Cell Physiol.

[B28] Hoffmann A, Hammes E, Plieth C, Desel C, Sattelmacher B, Hansen UP (2005). Effect of CO_2 _supply on formation of reactive oxygen species in *Arabidopsis thaliana*. Protoplasma.

[B29] Nakagami H, Soukupova H, Schikora A, Zarsky V, Hirt H (2006). A mitogen-activated protein kinase kinase kinase mediates reactive oxygen species homeostasis in Arabidopsis. J Biol Chem.

[B30] Kalbina I, Strid A (2006). Supplementary ultraviolet-B irradiation reveals differences in stress responses between *Arabidopsis thaliana *ecotypes. Plant Cell Environ.

[B31] Flors C, Fryer MJ, Waring J, Reeder B, Bechtold U, Mullineaux PM, Nonell S, Wilson MT, Baker NR (2006). Imaging the production of singlet oxygen in vivo using a new fluorescent sensor, Singlet Oxygen Sensor Green^®^. J Exp Bot.

[B32] Fryer MJ, Oxborough K, Mullineaux PM, Baker NR (2002). Imaging of photo-oxidative stress responses in leaves. J Exp Bot.

[B33] Thordal-Christensen H, Yangdou Wei ZZ, Collinge DB (1997). Subcellular localization of H_2_O_2 _in plants. H_2_O_2 _accumulation in papillae and hypersensitive response during the barley-powdery mildew interaction. Plant J.

[B34] Huckelhoven R, Fodor J, Trujillo M, Kogel KH (2000). Barley Mla and Rar mutants compromised in the hypersensitive cell death response against *Blumeria graminis *f.sp *hordei *are modified in their ability to accumulate reactive oxygen intermediates at sites of fungal invasion. Planta.

[B35] Lee BH, Lee H, Xiong L, Zhu JK (2002). A mitochondrial complex I defect impairs cold-regulated nuclear gene expression. Plant Cell.

[B36] Dutilleul C, Garmier M, Noctor G, Mathieu C, Chetrit P, Foyer CH, de Paepe R (2003). Leaf mitochondria modulate whole cell redox homeostasis, set antioxidant capacity, and determine stress resistance through altered signaling and diurnal regulation. Plant Cell.

[B37] Laloi C, Mestres-Ortega D, Marco Y, Meyer Y, Reichheld JP (2004). The Arabidopsis cytosolic thioredoxin *h5 *gene induction by oxidative stress and its W-box-mediated response to pathogen elicitor. Plant Physiol.

[B38] Marchand C, Le Marechal P, Meyer Y, Miginiac-Maslow M, Issakidis-Bourguet E, Decottignies P (2004). New targets of Arabidopsis thioredoxins revealed by proteomic analysis. Proteomics.

[B39] Wong JH, Balmer Y, Cai N, Tanaka CK, Vensel WH, Hurkman WJ, Buchanan BB (2003). Unraveling thioredoxin-linked metabolic processes of cereal starchy endosperm using proteomics. FEBS Letters.

[B40] Yamazaki D, Motohashi K, Kasama T, Hara Y, Hisabori T (2004). Target proteins of the cytosolic thioredoxins in *Arabidopsis thaliana*. Plant Cell Physiol.

[B41] Das KC, Das CK (2000). Thioredoxin, a singlet oxygen quencher and hydroxyl radical scavenger: Redox independent functions. Biochem Biophys Res Commun.

[B42] Havaux M, Dall'Osto L, Bassi R (2007). Zeaxanthin has enhanced antioxidant capacity with respect to all other xanthophylls in Arabidopsis leaves and functions independent of binding to PSII antennae. Plant Physiol.

[B43] Fryer MJ (1992). The antioxidant effects of thylakoid vitamin-E (alpha-tocopherol). Plant Cell Environ.

[B44] Havaux M, Eymery F, Porfirova S, Rey P, Dormann P (2005). Vitamin E protects against photoinhibition and photooxidative stress in *Arabidopsis thaliana*. Plant Cell.

[B45] Matysik J, Alia, Bhalu B, Mohanty P (2002). Molecular mechanisms of quenching of reactive oxygen species by proline under stress in plants. Curr Sci.

[B46] Kliebenstein DJ, Monde RA, Last RL (1998). Superoxide dismutase in Arabidopsis: An eclectic enzyme family with disparate regulation and protein localization. Plant Physiol.

[B47] Apel K, Hirt H (2004). Reactive oxygen species: Metabolism, oxidative stress, and signal transduction. Annu Rev Plant Biol.

[B48] Noctor G, Foyer CH (1998). Ascorbate and glutathione: Keeping active oxygen under control. Annu Rev Plant Physiol Plant Mol Biol.

[B49] Willekens H, Chamnongpol S, Davey M, Schraudner M, Langebartels C, VanMontagu M, Inze D, VanCamp W (1997). Catalase is a sink for H_2_O_2 _and is indispensable for stress defence in C-3 plants. EMBO J.

[B50] Laloi C, Przybyla D, Apel K (2006). A genetic approach towards elucidating the biological activity of different reactive oxygen species in *Arabidopsis thaliana*. J Exp Bot.

[B51] Scott I, Logan DC (2008). Mitochondrial morphology transition is an early indicator of subsequent cell death in Arabidopsis. New Phytol.

[B52] Asada K, Kiso K, Yoshikawa K (1974). Univalent reduction of molecular oxygen by spinach chloroplasts on illumination. J Biol Chem.

[B53] Couée I, Sulmon C, Gouesbet G, El Amrani A (2006). Involvement of soluble sugars in reactive oxygen species balance and responses to oxidative stress in plants. J Exp Bot.

[B54] Brouquisse R, James F, Raymond P, Pradet A (1991). Study of glucose starvation in excised maize root-tips. Plant Physiol.

[B55] Hooks MA, Bode K, Couee I (1995). Regulation of acyl-coa oxidases in maize seedlings. Phytochemistry.

[B56] Däschner K, Couée I, Binder S (2001). The mitochondrial isovaleryl-coenzyme A dehydrogenase of Arabidopsis oxidizes intermediates of leucine and valine catabolism. Plant Physiol.

[B57] Batish DR, Singh HP, Setia N, Kaur S, Kohli RK (2006). 2-Benzoxazolinone (BOA) induced oxidative stress, lipid peroxidation and changes in some antioxidant enzyme activities in mung bean (*Phaseolus aureus*). Plant Physiol Biochem.

[B58] Shao N, Krieger-Liszkay A, Schroda M, Beck CF (2007). A reporter system for the individual detection of hydrogen peroxide and singlet oxygen: its use for the assay of reactive oxygen species produced *in vivo*. Plant J.

[B59] Chao L, Zhou QX, Chen S, Cui S, Wang ME (2007). Single and joint stress of acetochlor and Pb on three agricultural crops in northeast China. J Environ Sci (China).

[B60] Tewari R, Hahn E-J, Paek K-Y (2008). Modulation of copper toxicity-induced oxidative damage by nitric oxide supply in the adventitious roots of Panax ginseng. Plant Cell Reports.

[B61] Orozco-Cardenas M, Ryan CA (1999). Hydrogen peroxide is generated systemically in plant leaves by wounding and systemin via the octadecanoid pathway. Proc Natl Acad Sci USA.

[B62] Laloi C, Apel K, Danon A (2004). Reactive oxygen signalling: the latest news. Curr Opin Plant Biol.

[B63] Laloi C, Stachowiak M, Pers-Kamczyc E, Warzych E, Murgia I, Apel K (2007). Cross-talk between singlet oxygen- and hydrogen peroxide-dependent signaling of stress responses in *Arabidopsis thaliana*. Proc Natl Acad Sci USA.

[B64] Gadjev I, Vanderauwera S, Gechev TS, Laloi C, Minkov IN, Shulaev V, Apel K, Inze D, Mittler R, Van Breusegem F (2006). Transcriptomic footprints disclose specificity of reactive oxygen species signaling in Arabidopsis. Plant Physiol.

[B65] op den Camp RGL, Przybyla D, Ochsenbein C, Laloi C, Kim CH, Danon A, Wagner D, Hideg E, Gobel C, Feussner I (2003). Rapid induction of distinct stress responses after the release of singlet oxygen in Arabidopsis. Plant Cell.

[B66] Jimenez A, Hernandez JA, delRio LA, Sevilla F (1997). Evidence for the presence of the ascorbate-glutathione cycle in mitochondria and peroxisomes of pea leaves. Plant Physiol.

[B67] Davletova S, Rizhsky L, Liang HJ, Zhong SQ, Oliver DJ, Coutu J, Shulaev V, Schlauch K, Mittler R (2005). Cytosolic ascorbate peroxidase 1 is a central component of the reactive oxygen gene network of Arabidopsis. Plant Cell.

[B68] Moon H, Baek D, Lee B, Prasad DT, Lee SY, Cho MJ, Lim CO, Choi MS, Bahk J, Kim MO (2002). Soybean ascorbate peroxidase suppresses Bax-induced apoptosis in yeast by inhibiting oxygen radical generation. Biochem Biophys Res Commun.

[B69] Yoshida S, Tamaoki M, Shikano T, Nakajima N, Ogawa D, Ioki M, Aono M, Kubo A, Kamada H, Inoue Y (2006). Cytosolic dehydroascorbate reductase is important for ozone tolerance in *Arabidopsis thaliana*. Plant Cell Physiol.

[B70] Chung J-S, Zhu J-K, Bressan RA, Hasegawa PM, Shi H (2008). Reactive oxygen species mediate Na+-induced *SOS1 *mRNA stability in Arabidopsis. Plant J.

[B71] Xiong Y, Contento AL, Bassham DC (2007). Disruption of autophagy results in constitutive oxidative stress in Arabidopsis. Autophagy.

[B72] Lichtenthaler HK, Wellburn AR (1983). Determinations of total carotenoids and chlorophylls a and b of leaf extracts in different solvents. Biochem Soc Trans.

[B73] http://rsb.info.nih.gov/ij/index.html.

[B74] Rao MV, Davis KR (1999). Ozone-induced cell death occurs via two distinct mechanisms in Arabidopsis: the role of salicylic acid. Plant J.

[B75] Rook GA, Steele J, Umar S, Dockrell HM (1985). A simple method for the solubilisation of reduced NBT, and its use as a colorimetric assay for activation of human macrophages by gamma-interferon. J Immunol Methods.

[B76] Mookerjee A, Basu JM, Majumder S, Chatterjee S, Panda GS, Dutta P, Pal S, Mukherjee P, Efferth T, Roy S (2006). A novel copper complex induces ROS generation in doxorubicin resistant *Ehrlich ascitis *carcinoma cells and increases activity of antioxidant enzymes in vital organs *in vivo*. BMC Cancer.

[B77] Kotchoni SO, Kuhns C, Ditzer A, Kirch HH, Bartels D (2006). Over-expression of different aldehyde dehydrogenase genes in *Arabidopsis thaliana *confers tolerance to abiotic stress and protects plants against lipid peroxidation and oxidative stress. Plant Cell Environ.

[B78] Beauchamp C, Fridovich I (1971). Superoxide dismutase: improved assays and an assay applicable to acrylamide gels. Anal Biochem.

[B79] Nakano Y, Asada K (1981). Hydrogen-peroxide is scavenged by ascorbate-specific peroxidase in spinach-chloroplasts. Plant Cell Physiol.

[B80] Hossain MA, Asada K (1984). Purification of dehydroascorbate reductase from spinach and its characterization as a thiol enzyme. Plant Cell Physiol.

[B81] Hossain MA, Nakano Y, Asada K (1984). Monodehydroascorbate reductase in spinach-chloroplasts and its participation in regeneration of ascorbate for scavenging hydrogen-peroxide. Plant Cell Physiol.

[B82] Smith IK, Vierheller TL, Thorne CA (1988). Assay of glutathione-reductase in crude tissue-homogenates using 5,5'-dithiobis(2-nitrobenzoic acid). Anal Biochem.

[B83] Aebi H (1984). Catalase *in vitro*. Methods Enzymol.

[B84] http://www.ebi.ac.uk/arrayexpress/.

[B85] Lurin C, Andres C, Aubourg S, Bellaoui M, Bitton F, Bruyere C, Caboche M, Debast C, Gualberto J, Hoffmann B (2004). Genome-wide analysis of Arabidopsis pentatricopeptide repeat proteins reveals their essential role in organelle biogenesis. Plant Cell.

