# Supplementary figures and images for: Differential patterns of reactive oxygen species and antioxidative mechanisms during atrazine injury and sucrose-induced tolerance in Arabidopsis thaliana plantlets
Source: BMC Plant Biol. 2009 Mar 13;9:28. doi: 10.1186/1471-2229-9-28 (PMC2661893; doi:10.1186/1471-2229-9-28)

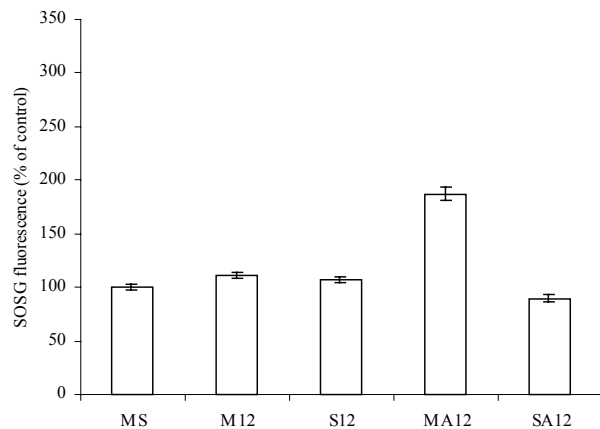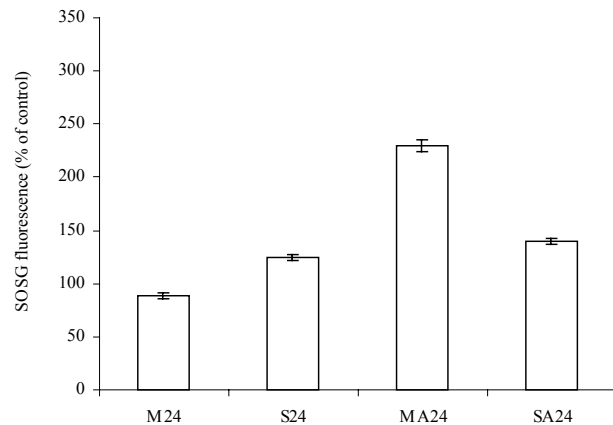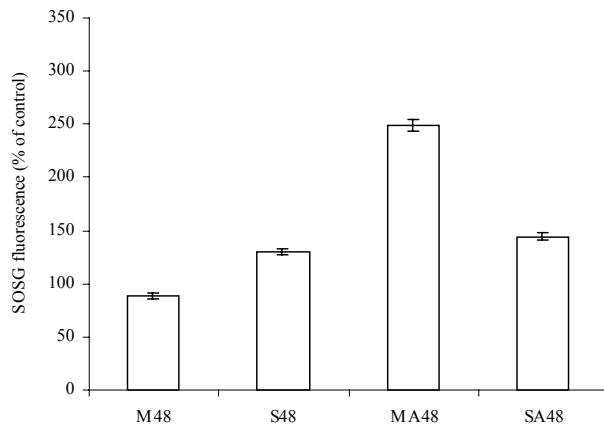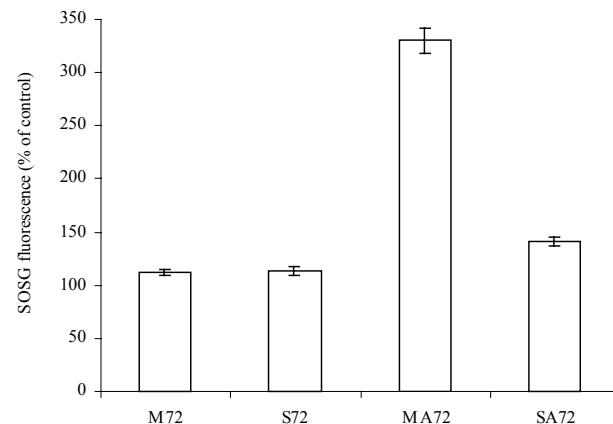

Supplement: Additional file 1 — Singlet oxygen detections using the SOSG probe have been done on 3-week-old MS-grown Arabidopsis thaliana plantlets subjected to subsequent treatment (12, 24, 48 or 72 hours) with 80 mM mannitol (M), 80 mM sucrose (S), 80 mM mannitol plus 10 μM atrazine (MA) or 80 mM sucrose plus 10 μM atrazine (SA). Image analysis and quantification of fluorescence was performed using ImageJ software. Changes in average intensities are shown as percentage of mean fluorescence intensity of MS-grown plantlets as control. [file 1471-2229-9-28-S1.pdf]

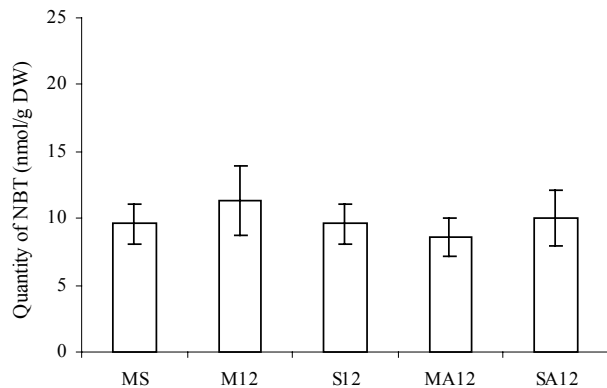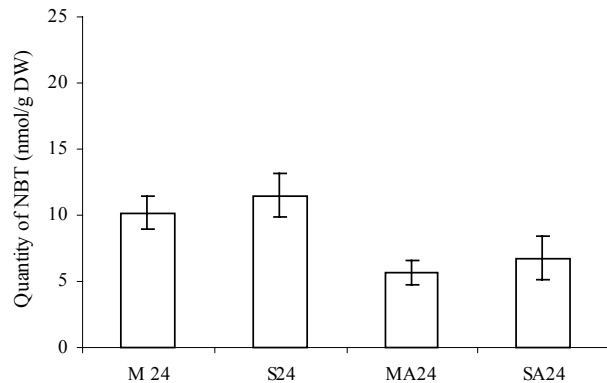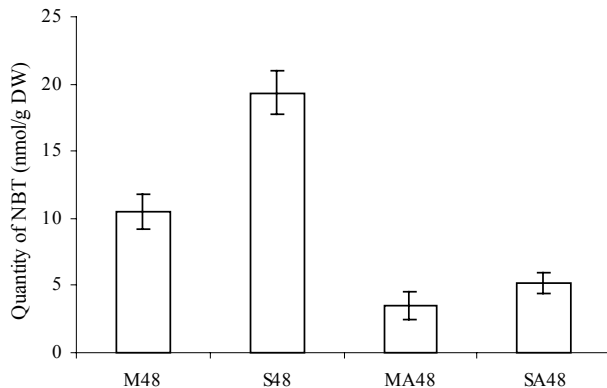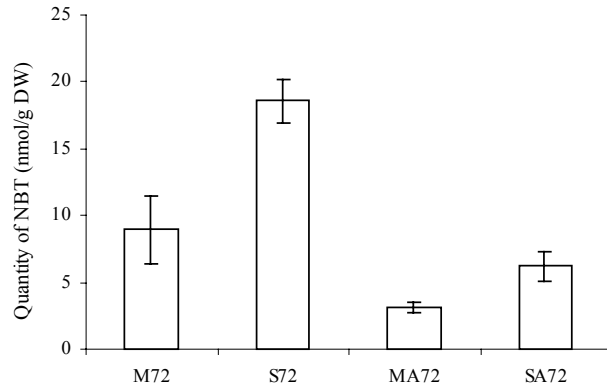

Supplement: Additional file 2 — Detections and quantification have been done on 3-week-old MS-grown Arabidopsis thaliana plantlets subjected to subsequent treatment (12, 24, 48 or 72 hours) with 80 mM mannitol (M), 80 mM sucrose (S), 80 mM mannitol plus 10 μM atrazine (MA) or 80 mM sucrose plus 10 μM atrazine (SA). Superoxide radical content was expressed as nmoles of reduced NBT per g DW. [file 1471-2229-9-28-S2.pdf]

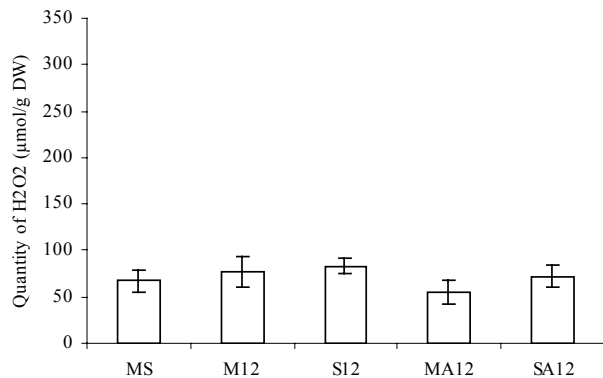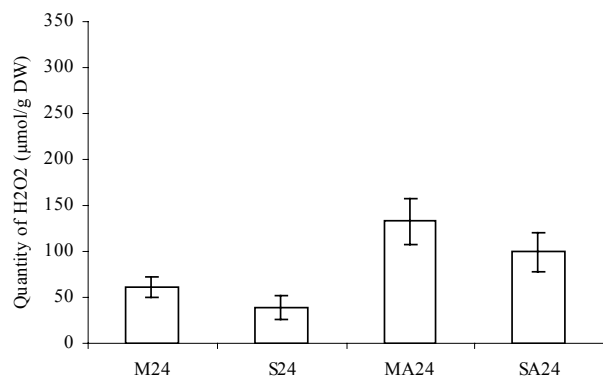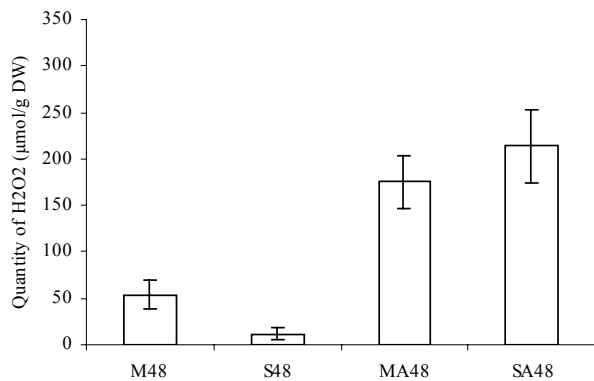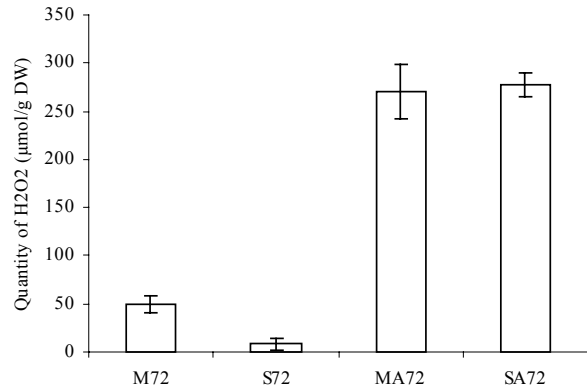

Supplement: Additional file 3 — Detections and quantification have been done on 3-week-old MS-grown Arabidopsis thaliana plantlets subjected to subsequent treatment (12, 24, 48 or 72 hours) with 80 mM mannitol (M), 80 mM sucrose (S), 80 mM mannitol plus 10 μM atrazine (MA) or 80 mM sucrose plus 10 μM atrazine (SA). Hydrogen peroxide content was expressed as μ moles of H2O2 per g DW. [file 1471-2229-9-28-S3.pdf]
